# Augmented Respiratory–Sympathetic Coupling and Hemodynamic Response to Acute Mild Hypoxia in Female Rodents With Chronic Kidney Disease

**DOI:** 10.3389/fphys.2021.623599

**Published:** 2021-05-25

**Authors:** Manash Saha, Qi-Jian Sun, Cara M. Hildreth, Peter G. R. Burke, Jacqueline K. Phillips

**Affiliations:** ^1^Department of Biomedical Sciences, Macquarie University, Sydney, NSW, Australia; ^2^Department of Nephrology, National Institute of Kidney Disease and Urology, Dhaka, Bangladesh; ^3^Graduate School of Medicine, Wollongong University, Wollongong, NSW, Australia; ^4^Neuroscience Research Australia, Sydney, NSW, Australia

**Keywords:** female, respiratory sympathetic modulation, chronic kidney disease, hypertension, chemoreflex, hypoxia, hypercapnia

## Abstract

Carotid body feedback and hypoxia may serve to enhance respiratory–sympathetic nerve coupling (respSNA) and act as a driver of increased blood pressure. Using the Lewis polycystic kidney (LPK) rat model of chronic kidney disease, we examined respSNA in adult female rodents with CKD and their response to acute hypoxia or hypercapnia compared to Lewis control animals. Under urethane anesthesia, phrenic nerve activity, splanchnic sympathetic nerve activity (sSNA), and renal sympathetic nerve activity (rSNA) were recorded under baseline conditions and during mild hypoxic or hypercapnic challenges. At baseline, tonic SNA and blood pressure were greater in female LPK rats versus Lewis rats (all *P* < 0.05) and respSNA was at least two-fold larger [area under the curve (AUC), sSNA: 7.8 ± 1.1 vs. 3.4 ± 0.7 μV s, rSNA: 11.5 ± 3 vs. 4.8 ± 0.7 μV s, LPK vs. Lewis, both *P* < 0.05]. Mild hypoxia produced a larger pressure response in LPK [Δ mean arterial pressure (MAP) 30 ± 6 vs. 12 ± 6 mmHg] and augmented respSNA (ΔAUC, sSNA: 8.9 ± 3.4 vs. 2 ± 0.7 μV s, rSNA: 6.1 ± 1.2 vs. 3.1 ± 0.7 μV s, LPK vs. Lewis, all *P* ≤ 0.05). In contrast, central chemoreceptor stimulation produced comparable changes in blood pressure and respSNA (ΔMAP 13 ± 3 vs. 9 ± 5 mmHg; respSNA ΔAUC, sSNA: 2.5 ± 1 vs. 1.3 ± 0.7 μV s, rSNA: 4.2 ± 0.9 vs. 3.5 ± 1.4 μV s, LPK vs. Lewis, all *P* > 0.05). These results demonstrate that female rats with CKD exhibit heightened respSNA coupling at baseline that is further augmented by mild hypoxia, and not by hypercapnia. This mechanism may be a contributing driver of hypertension in this animal model of CKD.

## Introduction

Sympathetic nerve activity (SNA) varies across different phases of the respiratory cycle. This harmonious relationship is termed respiratory sympathetic modulation or coupling (respSNA) (Haselton and Guyenet, 1989; Czyzyk-Krzeska and Trzebski, 1990; Dick et al., 2004) and is an important homeostatic mechanism that allows synchronization between the cardiovascular and respiratory systems. Respiratory sympathetic coupling can change in response to metabolic challenges such as hypoxia, with peripheral chemoreceptor activation inducing reflex respiratory and autonomic adjustments (Dick et al., 2004; Mandel and Schreihofer, 2009). For instance, hypoxia can generate an active expiratory motor pattern that is coupled to increased sympathetic activity (Moraes et al., 2014). Augmentation of respSNA has been linked explicitly to sympathetic hyperactivity and hypertension in animal models of both primary and secondary hypertension (Simms et al., 2009; Toney et al., 2010; Zoccal and Machado, 2010).

In patients with chronic kidney disease (CKD), high blood pressure is a common comorbidity, and sympathetic hyperactivity is believed to contribute to their hypertensive state and increased risk of cardiovascular morbidity (Tonelli et al., 2006; Hering et al., 2007; Rubinger et al., 2012; Salman et al., 2014; Santoro and Mandreoli, 2014). Studies utilizing chemoreflex deactivation with 100% oxygen in CKD patients support the hypothesis that tonic activation of peripheral chemoreceptors contributes to this elevated sympathetic activity (Hering et al., 2007). The concept of tonic activation of peripheral chemoreceptors in CKD aligns with chronic systemic hypoxia in CKD patients, which has been proposed to be mediated by a combination of vascular changes and anemia (Prommer et al., 2018). Increased tonicity and sensitivity of carotid body afferents are documented in human and mammalian models of hypertension (Tan et al., 2010; Abdala et al., 2012; Sinski et al., 2012; Paton et al., 2013; Moraes et al., 2018), heart failure (Marcus et al., 2014, 2018), human sleep apnea (Narkiewicz et al., 1998, 1999), and other cardiovascular diseases and are a causal mechanism for the observed increase in SNA.

We have previously shown an association between respSNA and sympathetic hyperactivity in adult male Lewis polycystic kidney (LPK) rats, a genetic CKD rat model of secondary hypertension (Saha et al., 2019). We demonstrated in these animals that peripheral chemoreceptor activation evoked a larger increase in respSNA compared to control animals (Saha et al., 2019), consistent with rats who have been exposed to conditions of chronic hypoxia, which present an enhanced pattern of central sympathetic–respiratory coupling (Zoccal et al., 2008).

Population-based studies suggest that female CKD patients may have a reduced risk of cardiovascular morbidity and mortality compared to male patients (Franczyk-Skora et al., 2012; Nitsch et al., 2013). Noting that sex differences exist in autonomic, cardiovascular, and ventilatory responses to cardiorespiratory reflex activation in healthy individuals (Crofton et al., 1988; Chen and DiCarlo, 1996; Tank et al., 2005; Kim et al., 2011; Joshi and Edgell, 2019), this sexual dimorphism may be a contributing pathophysiological mechanism to the apparent protective impact of female sex on cardiovascular disease risk. For example, we have previously demonstrated that female LPK show a lesser degree of hypertension compared to male animals (Phillips et al., 2007) and that the mechanism underpinning sympathetic nerve and heart rate (HR) baroreflex dysfunction operates differently between males and females (Salman et al., 2014, 2015b), with male animals demonstrating reduced baroreflex control due to disturbances in both afferent and central baroreflex processing, whereas female animals demonstrate altered processing within the central component of the baroreflex only. Further, sex differences exist within rodents in the profile of respSNA after exposure to chronic intermittent hypoxia, with both sexes showing increased SNA modulation, but female rats exhibiting a change during inspiration, whereas for male rats, it is during expiration (Souza et al., 2016, 2017). To our knowledge, it is unknown whether sex differences exist in either the pattern of respSNA or the response to respiratory challenge in CKD. This is important to study in order to provide completeness of representation of sex in biomedical research studies (Beery and Zucker, 2011) as different therapeutic approaches may need to be considered if there is an underlying mechanistic difference in the disease state.

The present study therefore was designed to characterize the pattern of respiratory-related SNA in female rodents with CKD and assess changes in respSNA and hemodynamic responses induced by chemoreceptor stimulation, comparing responses to peripheral or central CO_2_ chemoreception, thereby allowing determination of the key components of the integrated chemoreflex response to be considered. Our primary hypothesis was that the CKD-related hypertensive female rats would exhibit both heightened sympathetic–respiratory coupling and responsiveness to hypoxic chemoreceptor stimulation when compared to normotensive controls. Based on our previous demonstration of a milder hypertensive phenotype and that afferent baroreceptor function is unaffected in the female LPK, we further hypothesized that the cardiorespiratory reactivity to hypoxic chemoreceptor stimulation would be milder in females with CKD relative to male CKD animals examined under the same experimental conditions.

## Materials and Methods

All procedures were approved by the Animal Ethics Committee of Macquarie University, Sydney, New South Wales, Australia, and adhered to the Australian Code of Practice for the Care and Use of Animals for Scientific Purposes. Adult female LPK rats aged 12 to 13 weeks (*n* = 8) and age-matched control female Lewis rats (*n* = 9) were used. Stage of estrus cycle was not determined in the female animals. Animals were obtained from the Animal Resources Center, Perth, Western Australia. Animals arrived 2 weeks prior to experiments. During this period, animals underwent the process of acclimatization to a new housing environment and experimental procedures, including training for metabolic cage placement for 24-h urine collection. During this period, animals were monitored for normal weight and growth for their age. The male data to which the female results are compared (Supplementary Tables 1–5) have been published prior (Saha et al., 2019). The male and female experiments were undertaken in parallel under the same experimental conditions, with animals acquired from the same source, held in the same facility and physiological data acquired using the same equipment. The data from the female animals have not been previously published.

### Renal Function

Urine samples over a 24-h period were collected from all animals 48 h before experimentation. Urine volume, urinary creatinine, and protein levels were determined using an IDEXX Vetlab analyzer (IDEXX Laboratories Pty Ltd., Sydney, New South Wales, Australia). An arterial blood sample was collected on the day of the experiment to determine plasma urea and creatinine. An estimate of creatinine clearance was also calculated (Yao et al., 2015).

### Surgical Procedures

Anesthesia was induced with 10% (wt/vol) ethyl carbamate (Urethane; Sigma–Aldrich, St. Louis, MO, United States) dissolved in 0.9% NaCl solution at a dose of 1.3 g/kg delivered intraperitoneally (i.p.). Anesthetic depth was assessed by periodic hind-paw pinch and supplemental doses of urethane given [65 mg/kg i.p. before catheterization and then intravenous (i.v.)] as required. Body temperature was maintained at 37°C ± 0.5°C using a homeothermic heating blanket (Harvard Apparatus, Holliston, MA, United States) and infrared heating lamp. The right femoral artery was cannulated for measurement of arterial pressure (AP) and for measurements of arterial blood chemistry. The femoral vein cannulated for administration of Ringer’s lactate, 5 mL/kg per hour. The arterial cannula was connected to pressure transducer. The AP signal was passed through a bioamplifier, digitized, and sampled at 200 Hz using a CED 1401 with Spike 2 software (Cambridge Electronic Designs Ltd., Cambridge, United Kingdom). A tracheostomy was performed, and an endotracheal tube secured in place to permit mechanical ventilation. A bilateral vagotomy was performed to eliminate lung stretch receptor afferents entraining central inspiratory activity. Animals were ventilated with O_2_-enriched room air (7025 Rodent Ventilator; Ugo Basile, Gemonio, Italy) and paralyzed with pancuronium bromide (induction: 2 mg/kg i.v.; maintenance; 1 mg/kg) (AstraZeneca, Australia). The left phrenic nerve was approached dorsally, isolated, tied with silk thread, and cut distal to the tie (Burke et al., 2010). The left splanchnic sympathetic and renal sympathetic nerves were dissected using a retroperitoneal approach as previously described (Burke et al., 2011; Saha et al., 2019). All nerves were bathed in a liquid paraffin pool, and the central end recorded using bipolar silver wire recording electrodes, amplified, and bandpass-filtered (100–3,000 Hz) with a bioamplifier (CWE Inc., Ardmore, PA, United States) and sampled at 5 kHz using a CED 1401 plus and Spike 2 software. All recordings were calibrated to a presetting 50 μV using the same bioamplifier for all experiments.

The preparation was then stabilized for 30 min and arterial blood collected and analyzed for SaO_2_, pH, PCO_2_, and HCO_3_ (VetStat Electrolyte and arterial blood gas analyzer; IDEXX Laboratories Pty Ltd.). SaO_2_ was used to measure the oxygenation of blood instead of PaO_2_ as SaO_2_ and is directly relatable to human studies (Hering et al., 2007). If required, arterial blood chemistry status was adjusted to maintain parameters within the following range: pH 7.4 ± 0.05, PCO_2_ = 40 ± 5 mmHg, HCO_3_^–^ = 24 ± 2 mmol/L, SaO_2_ = 100%, and end-tidal CO_2_ = 4.5% ± 0.5%. This was achieved by adjusting the rate and depth of the mechanical ventilator and/or slow bolus of 5% sodium bicarbonate. A second arterial blood gas analysis was undertaken as required.

### Experimental Protocol

The experimental protocol followed was as we have published previously (Saha et al., 2019). Briefly, following stabilization, the integrity of recordings of the splanchnic and renal nerve activity (sSNA and rSNA, respectively) was confirmed by SNA pulse modulation and demonstration of a baroreflex response to a bolus injection of phenylephrine (50 μg/kg i.v.; Sigma–Aldrich). Baseline recordings were then established under control conditions for 30 min. As documented in male animals (Saha et al., 2019), mild hypoxia was induced by switching animals to room air, without oxygen supplementation, for 3 min without any change in ventilator rate or volume, as confirmed by arterial blood gas analysis (range of 82–84% SaO_2_). After hypoxic exposure, the preparation recovered for 30 min before a hypercapnic challenge of 5% carbogen (5% CO_2_ in 95% O_2_) for 3 min without any change in the ventilator rate or volume, as confirmed by arterial blood gas analyses (range of 65 ± 8 mmHg PCO_2_).

At completion of the study, animals were euthanized with 3 M potassium chloride delivered i.v. The electrical noise levels for phrenic nerve activity (PNA), sSNA, and rSNA were recorded and later subtracted in the data analysis.

### Data Analysis

Acquired data were analyzed offline in Spike 2. Mean arterial pressure (MAP), systolic blood pressure (SBP), diastolic blood pressure (DBP), pulse pressure (PP), and HR were determined. The splanchnic and renal sympathetic recordings were rectified and smoothed 0.1 s. PNA was rectified and smoothed 0.05 s. Baseline data for the analysis were defined as the 30-s period at the end of the control ventilation period immediately prior to the hypoxic and hypercapnic exposures. Response to each chemo challenge was analyzed over a 30-s period once PNA had stabilized for 1 to 2 min. Responses are expressed as a change (Δ) relative to the 30-s period immediately before each stimulus. The PNA amplitude, frequency (number cycle.min^–1^), and inspiratory duration (in seconds) were determined. Ensemble averages of rectified and smoothed rSNA and sSNA triggered to PNA were then generated from the last 30 s of the hypoxic or hypercapnia epoch. The respiratory cycle was divided into three phases: inspiratory (I), postinspiratory (PI) and late expiratory (E) based on well-established features of the respiratory motor pattern (Bautista et al., 2010; Burke et al., 2010, 2015). For the renal and splanchnic SNA, the mean level was measured from the period 200 ms before the onset of the phrenic burst corresponding to the late-expiratory E phase, and this was considered the baseline for all other measurements. From the ensemble averages, the following parameters were calculated: peak SNA activity (PA [μV]) being the maximal SNA burst coincident with inspiratory/PI phase, the duration [from onset of excitatory activity to return to baseline (in seconds)], and area under the curve (AUC) of respSNA excitatory peak (μV s) determined as the integral of the waveform. AUCs of respSNA for I, PI, and E phase of the phrenic cycle were also calculated from the integral of the waveform.

### Statistical Analysis

Data are presented as mean ± SEM. Unpaired two-tailed Student *t* test was used to examine strain differences in renal function, cardiorespiratory function, and SNA. Two-way analysis of variance with repeated measures and Bonferroni corrections was used to examine strain differences in respiratory modulation of SNA under control conditions and in response to hypoxia or hypercapnia, with strain and ventilatory condition (i.e., normoxia, hypoxia, or hypercapnia) as variables. An unpaired two-tailed Student *t-*test was used to determine strain differences in delta changes from control to hypoxia or hypercapnia in cardiorespiratory function. Statistical analyses were performed with GraphPad Prism (GraphPad Software Inc., United States), and significance is indicated where *P* ≤ 0.05.

## Results

Female LPK animals exhibited elevated blood urea (LPK 23.7 ± 2.7 vs. Lewis 6.7 ± 0.5 mmol/L) and plasma creatinine (44.7 ± 7.9 vs. 13.1 ± 2.6 μmol/L) and reduced creatinine clearance (1.5 ± 0.3 vs. 7.4 ± 1.2 mL/min) (LPK n = 8 vs. Lewis n = 9; *P* < 0.001). This degree of renal dysfunction was not significantly different from that of male LPK animals of the same age [(Saha et al., 2019); Supplementary Table 1].

### Baseline Cardiorespiratory Parameters

**FIGURE 1:**
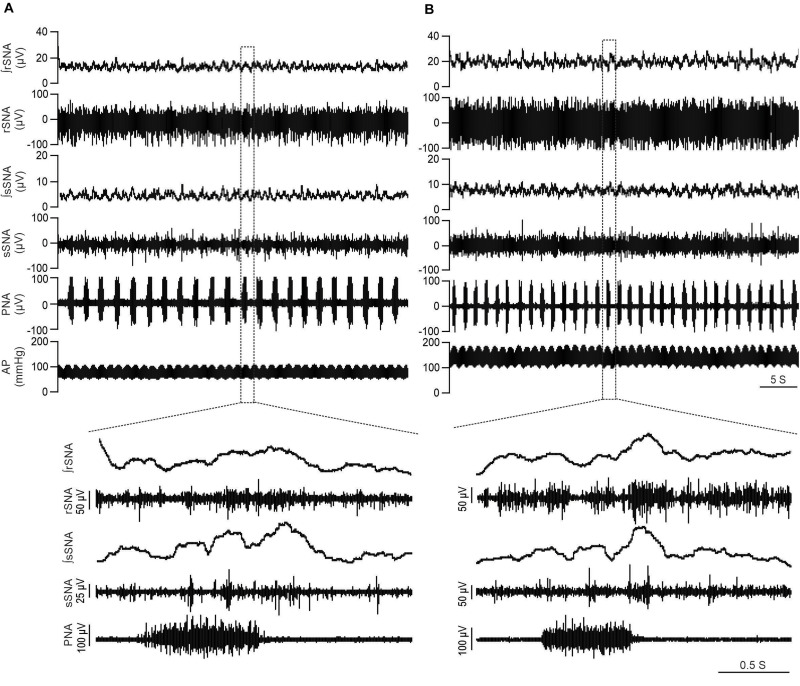
LPK rats exhibit higher SNA and BP in baseline recordings. Physiological recordings under urethane anesthesia representing raw and integrated renal sympathetic nerve activity (rSNA and ∫rSNA), splanchnic sympathetic nerve activity (sSNA and ∫sSNA), phrenic nerve activity (PNA) and arterial pressure (AP) in a 12-week-old Lewis rat **(A)** and 12-week-old LPK rat **(B)**. Note the higher rSNA, sSNA, and arterial pressure in the LPK in comparison to Lewis rats. Bottom panels illustrate Enlarged traces of one respiratory cycle with corresponding splanchnic and renal SNA.

Figure 1 illustrates individual recordings of SNA, PNA, and AP from a Lewis and LPK rat under baseline conditions. SBP, MAP, DBP, PP, HR, and SNA (both rSNA and sSNA) were elevated in the adult female LPK compared with age-matched Lewis controls (Table 1). In the adult female LPK, phrenic burst frequency was higher, and the duration of the phrenic burst shorter than the age- and arterial blood gas–matched Lewis controls (Table 1; *P* < 0.05). There was no significant difference in PNA amplitude (*P* = 0.23).

**TABLE 1:** Baseline cardiorespiratory function in female Lewis and LPK rats.

	Lewis	LPK
MAP (mmHg)	93 ± 4	126 ± 10*
SBP (mmHg)	125 ± 8	184 ± 18*
DBP (mmHg)	77 ± 4	99 ± 9*
PP (mmHg)	47 ± 8	84 ± 15*
HR (bpm)	427 ± 7	456 ± 37*
PNA amplitude (μV)	13.1 ± 3.5	20.9 ± 5.4
PNA frequency (cycles/min)	32 ± 1	42 ± 1*
PNA duration (s)	1.01 ± 0.05	0.69 ± 0.02*
rSNA (μV)	4.4 ± 0.8	7.5 ± 1.05*
sSNA (μV)	3.02 ± 0.5	6.4 ± 1.1*

Measures of cardiorespiratory parameters in Lewis and LPK rats. LPK, Lewis polycystic kidney; MAP, mean arterial pressure; SBP, systolic blood pressure; DBP, diastolic blood pressure; PP, pulse pressure; HR, heart rate; bpm, beats per minute; PNA, phrenic nerve amplitude; rSNA, renal sympathetic nerve activity; sSNA, splanchnic sympathetic nerve activity. Results are expressed as mean ± SEM. *P < 0.05 between Lewis rats and LPK rats as determined by Student *t*-test. *n* = 9 Lewis and *n* = 8 LPK except rSNA where *n* = 6 Lewis and *n* = 5 LPK.

**FIGURE 2:**
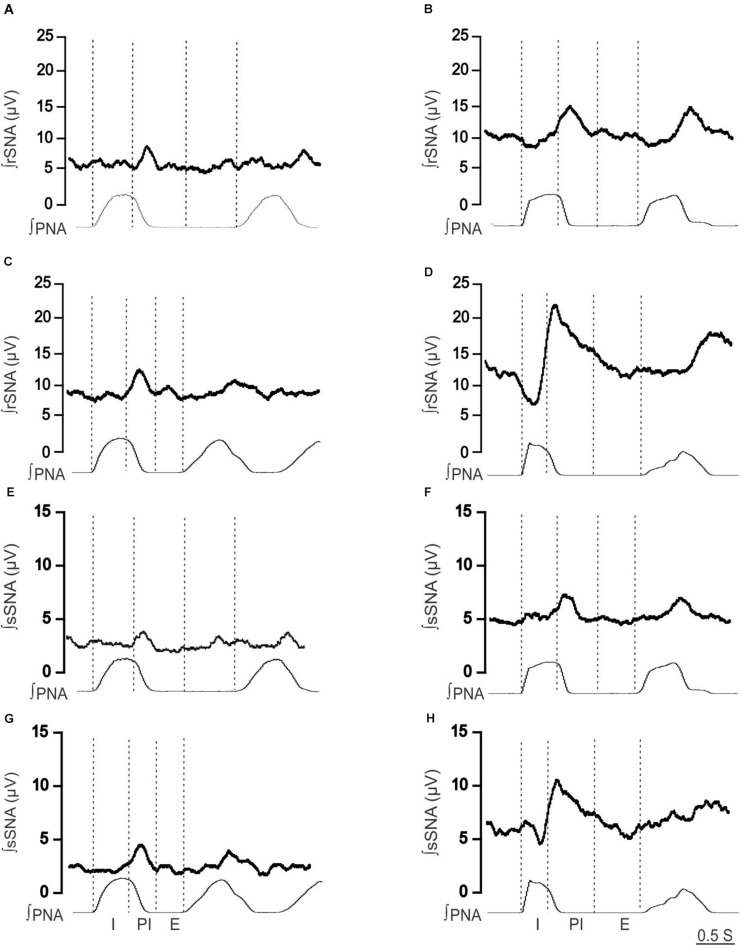
Effect of hypoxia on respiratory-related sympathetic nerve activity in adult female Lewis and LPK rats. Phrenic-triggered ensemble averages of renal sympathetic (∫rSNA) and splanchnic sympathetic nerve activity (∫sSNA) during different phases of the phrenic cycle (I, inspiration; PI, postinspiration; E, expiration) under control **(A,B,E,F)** and hypoxic conditions **(C,D,G,H)** in a Lewis rat **(A,C,E,G)** and LPK rat **(B,D,F,H)**.

To identify if respSNA was altered in female LPK, we first examined the temporal relationship between SNA and the respiratory cycle. Both sympathetic nerves exhibited respiratory sympathetic harmony, with a clear burst in SNA in the PI period observed in both Lewis and LPK rats (Figures 1, 2A,B,E,F, 4A,B,E,F). In Lewis rats, both sympathetic nerves showed weak PI coupling. In contrast, respSNA in the LPK exhibited inhibition during early I that was most obvious in rSNA (rSNA, I AUC, LPK vs. Lewis: −0.3 ± 0.1 vs. 0.2 ± 0.1 μV; *P* < 0.05; sSNA, I AUC, LPK vs. Lewis: 0.02 ± 0.05 vs. 0.2 ± 0.07 μV; *P* > 0.05) with a persistence of excitation in both rSNA and sSNA during the E period that was not different between strains (rSNA, E AUC, LPK vs. Lewis: 0.1 ± 0.04 vs. −0.03 ± 0.08 μV; *P* > 0.05; sSNA, E AUC, LPK vs. Lewis: 0.09 ± 0.06 vs. 0.03 ± 0.07 μV; *P* > 0.05).

Under control conditions, baseline respSNA in both nerves was significantly greater in the female LPK as reflected by a larger AUC (Table 2). This amplified respSNA was associated with both a higher magnitude of PA and longer duration of respSNA in LPK compared to Lewis control rats (Table 2).

**TABLE 2:** RespSNA parameters in splanchnic and renal nerves in adult female Lewis and LPK rats under baseline conditions.

		Lewis	LPK
PA (μV)	Splanchnic	4.0 ± 0.7	7.4 ± 1.3*
	Renal	5.9 ± 0.9	9.8 ± 1.5*
AUC	Splanchnic	3.4 ± 0.7	7.8 ± 1.1*
	Renal	4.8 ± 0.7	11.5 ± 3.0*
Duration (s)	Splanchnic	0.8 ± 0.08	1.1 ± 0.06*
	Renal	0.8 ± 0.06	1.1 ± 0.12*

Measures of respiratory–sympathetic coupling parameters for ∫sSNA and ∫rSNA in Lewis and LPK rats. LPK, Lewis polycystic kidney; PA, peak SNA activity (PA [μV]); AUC, area under the curve (s × μV), duration (of peak), and position (time from the onset of inspiration to PA). Results are expressed as mean ± SEM. *P ≤ 0.05 between Lewis rats and LPK rats as determined by Student *t*-test. *n* = 9 Lewis and *n* = 8 LPK for sSNA and *n* = 6 Lewis and *n* = 5 LPK for rSNA.

### Responses to Chemoreceptor Challenge

In the female LPK rats, peripheral chemoreceptor stimulation using a mild hypoxic challenge evoked a heightened pressor response compared to Lewis rats (Table 3). There was no significant difference in the increase in HR seen in both strains (Table 3). Hypoxia in the female LPK resulted in an increase in phrenic nerve amplitude, shortened burst duration, and slowed phrenic nerve frequency. The Lewis rats also responded to mild hypoxia with a comparable increase in phrenic nerve amplitude, a reduction of phrenic duration that was of a greater magnitude than that seen in the LPK, and a slight increase in frequency.

**TABLE 3:** Effects of peripheral or central chemoreceptor stimulation on cardiorespiratory pattern in adult female Lewis and LPK rats.

		Lewis (*n* = 9)	LPK (*n* = 8)
Hypoxia	Δ MAP (mmHg)	12 ± 6	30 ± 6*
	Δ SBP (mmHg)	15 ± 6	41 ± 8*
	Δ DBP (mmHg)	9 ± 5	27 ± 6*
	Δ PP (mmHg)	5 ± 1	15 ± 5*
	Δ HR (bpm)	25 ± 6	23 ± 3
	Δ PNA amplitude (μV)	7.3 ± 1.7	11.01 ± 1.9
	Δ PNA duration (s)	−0.2 ± 0.03	−0.07 ± 0.03*
	Δ PNA frequency (cycles/min)	5 ± 2	−10 ± 3*
Hypercapnia	Δ MAP (mmHg)	9 ± 5	13 ± 3
	Δ SBP (mmHg)	12 ± 6	20 ± 5
	Δ DBP (mmHg)	8 ± 4	10 ± 2
	Δ PP (mmHg)	3 ± 2	10 ± 3
	Δ HR (bpm)	−0.3 ± 1	2 ± 4
	Δ PNA amplitude (μV)	9.4 ± 2.8	9 ± 2.3
	Δ PNA duration (s)	0.8 ± 0.03	−0.06 ± 0.02*
	Δ PNA frequency (cycles/min)	−0.2 ± 1	−2 ± 1

Delta change (Δ) in phrenic triggered integrated phrenic nerve activity (PNA) and blood pressure (mmHg) in hypoxia (ventilated with only room air) or hypercapnia (ventilated with 5% CO_2_ with 95% O_2_) when switched from control condition (ventilated with room air enriched with 100% O_2_) in adult Lewis and LPK rats under urethane anesthesia. LPK, Lewis polycystic kidney; MAP, mean arterial pressure; SBP, systolic blood pressure; DBP, diastolic blood pressure; PP, pulse pressure. Results are expressed as mean ± SEM. *P ≤ 0.05 between Lewis rats and LPK rats as determined by Student *t*-test. *n* = number of animals per group.

**FIGURE 3:**
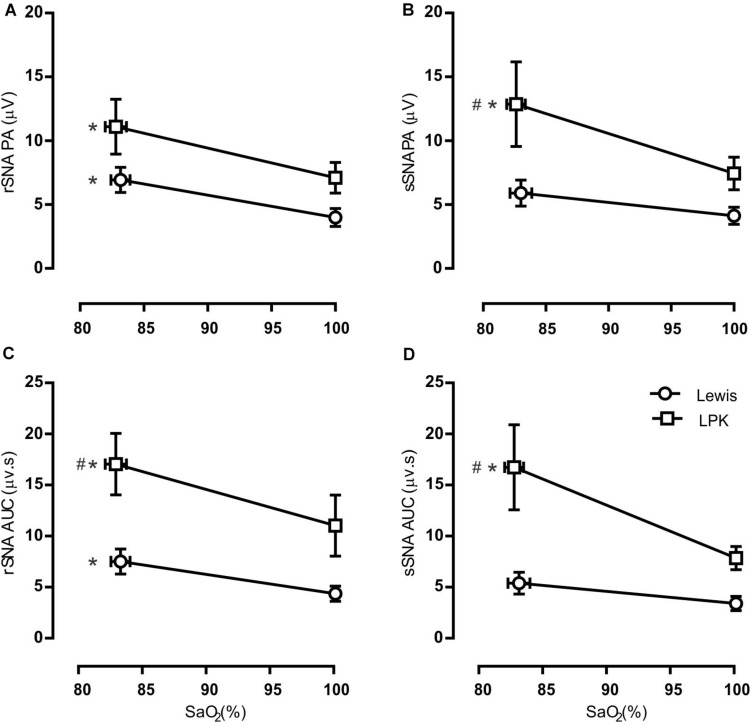
Group data of the effect of mild hypoxia on respiratory-related sympathetic nerve activity in adult female Lewis and LPK rats. Respiratory-related sympathetic nerve activity was measured as peak amplitude **(A,B)**, or area under the curve (AUC; **C,D**) of the phrenic triggered ∫rSNA **(A,C)** and ∫sSNA **(B,D)** during normoxia (control) and hypoxic conditions. Data are expressed as mean ± SEM *n* ≥ 5 per group. *P < 0.05 hypoxia versus baseline (normoxia) within each strain; #*P* < 0.05 LPK versus treatment-matched Lewis.

When comparing respSNA parameters after exposure to hypoxic conditions, the PA and AUC of respSNA of both rSNA and sSNA significantly increased in LPK rats, whereas in Lewis rats, the PA and AUC of respSNA of rSNA increased (Figures 2, 3). The increase in AUC in both rSNA and sSNA was greater in the LPK than that seen in Lewis rats Δ AUC, rSNA: LPK vs. Lewis: 6.07 ± 1.1 vs. 3.1 ± 0.7μV s, sSNA: LPK vs. Lewis: 8.9 ± 3.4 vs. 2 ± 0.7, both *P* ≤ 0.05). Of note was that under hypoxic conditions, in LPK rats, sympathoinhibition during inspiration increased significantly in rSNA (I AUC, rSNA, control vs. hypoxia: −0.25 ± 0.1 vs. −1.08 ± 0.2 μV, *P* ≤ 0.05*;* Figure 2). A comparable directional response was seen in sSNA in the LPK but did not reach significance (I AUC, sSNA, control vs. hypoxia: 0.02 ± 0.05 vs. −0.5 ± 0.1 μV, *P* = 0.08) (Figure 2). The AUC during E in the LPK was greater during hypoxia (E AUC, sSNA, control vs. hypoxia: 0.09 ± 0.06 vs. 0.75 ± 0.3 μV, *P* < 0.05), but there was no change in E AUC in the rSNA (*P* > 0.05). There was no change in I AUC or E AUC for either the renal or splanchnic nerves in the Lewis female rats (*P* > 0.05).

Mild hypercapnic stimuli produced a comparable increase in blood pressure between the strains (Table 3). Hypercapnia in the female LPK resulted in an increase in phrenic nerve amplitude, shortened burst duration, and slowed phrenic nerve frequency. The Lewis rats also responded to hypercapnia with an increase in phrenic nerve amplitude and reduced phrenic nerve frequency, which was not significantly different from that seen in the LPK. There was, however, an increased in phrenic duration as compared to the reduction seen in the LPK.

**FIGURE 4:**
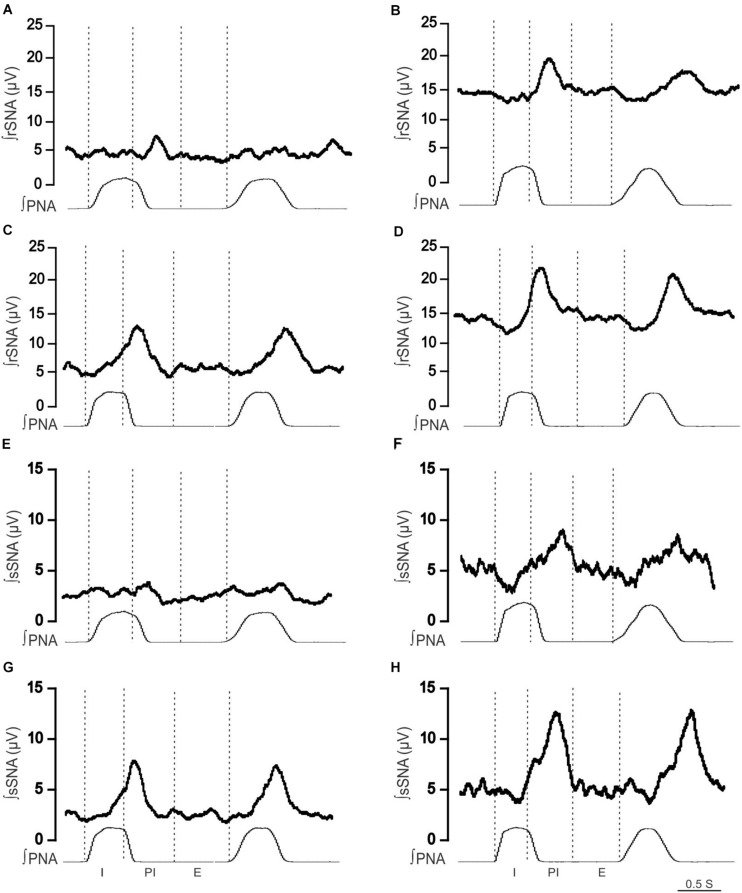
Effect of hypercapnia on respiratory-related sympathetic nerve activity in adult female Lewis and LPK rats. Phrenic-triggered ensemble averages of renal sympathetic (∫rSNA) and splanchnic sympathetic nerve activity (∫sSNA) during different phases of the phrenic cycle (I, inspiration; PI, postinspiration; E, expiration) under control **(A,B,E,F)** and hypercapnic conditions **(C,D,G,H)** in a Lewis rat **(A,C,E,G)** and LPK rat **(B,D,F,H)**.

**FIGURE 5:**
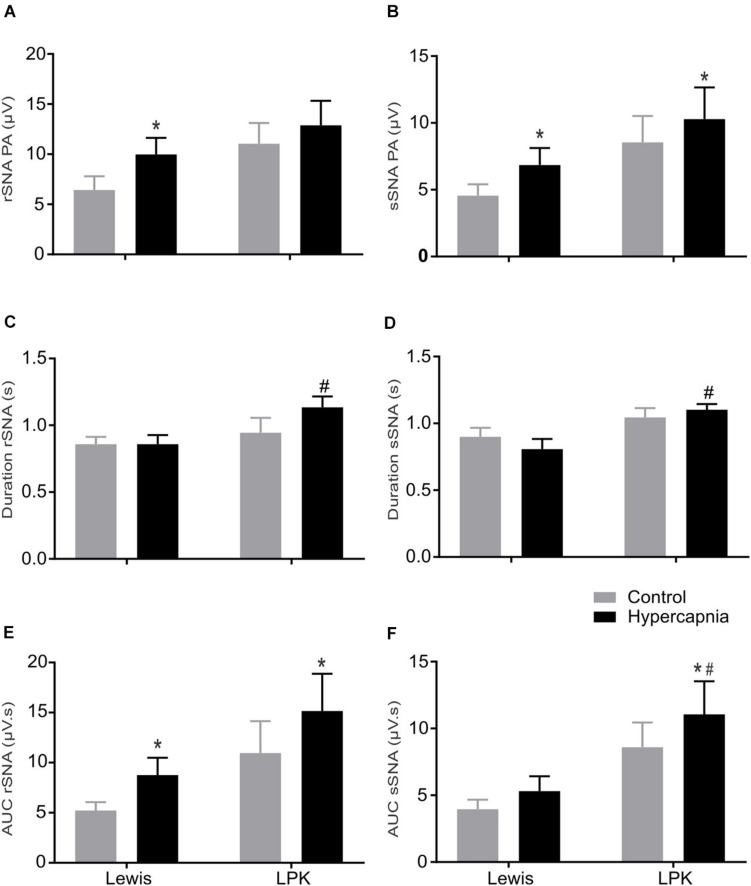
The effect of hypercapnia on respiratory-related sympathetic nerve activity in adult female Lewis and LPK rats: group data. Respiratory-related sympathetic nerve activity measured as peak amplitude **(A,B)**, duration **(C,D)**, or area under the curve (AUC; **E,F**) of the phrenic triggered ∫rSNA **(A,C,E)** and ∫sSNA **(B,D,F)** during normoxia (control) and hypercapnic conditions. Data are expressed as mean ± SEM *n* ≥ 5 per group. *P < 0.05 hypercapnia versus control within each strain; #*P* < 0.05 LPK versus treatment-matched Lewis.

When comparing respSNA parameters after exposure to hypercapnic conditions, in Lewis rats, both the renal and splanchnic nerves demonstrated an increase in PA, and in the renal nerve, there was also an increase in the measured AUC (Figures 4, 5). In the LPK, hypercapnia increased the AUC in both nerves, and there was also an increase in PA in the splanchnic nerve. Hypercapnia did not alter the duration of the curves in either strain, although it was greater in the LPK rats compared to Lewis rats under treatment conditions. The magnitude of observed changes in respSNA was comparable between the strains (Δ AUC, rSNA: LPK vs. Lewis: 4.2 ± 0.9 vs. 3.5 ± 1.4 sSNA: LPK vs. Lewis: 2.5 ± 1 vs. 1.3 ± 0.7μV s, both *P* > 0.05).

Sympathoinhibition during inspiration in the LPK did not significantly change in either rSNA or sSNA (I AUC, rSNA, control vs. hypercapnia: −0.21 ± 0.1 vs. −0.34 ± 0.1 sSNA: 0.04 ± 0.07 vs. 0.08 ± 0.09 μV s, both *P* > 0.05), and AUC in the E phase of respSNA for both nerves was comparable in response to hypercapnia in LPK rats (E AUC, rSNA, control vs. hypercapnia: 0.06 ± 0.03 vs. 0.04 ± 0.05 sSNA: 0.04 ± 0.03 vs. 0.04 ± 0.04 μV s, both *P* > 0.05). In Lewis rats, both nerves also showed no significant differences in inspiratory and expiratory period during hypercapnia (both *P* > 0.05).

### Female and Male LPK Rats Both Exhibit Augmented Respiratory–Sympathetic Coupling and Pressor Responses to Acute Mild Hypoxia and Hypercapnia

We have previously shown that when compared to normotensive control Lewis, male LPK rats are hypertensive and exhibit heightened respiratory-related sympathetic bursts at baseline, and mild hypoxia evokes larger increase in respSNA (Saha et al., 2019). The female rats in this study also exhibited a heightened pressor response and respSNA to both hypoxia and hypercapnia. Comparing our previous male data with the female data presented in this study, we observed no sex differences in the LPK or Lewis control rat in their pressor response or respSNA during hypoxia or hypercapnia. Comparison of renal functional data indicated that at the age studied, the degree of renal decline was also not significantly different (Supplementary Tables 1–5).

## Discussion

### Summary

The major findings of this study are that female rodents with CKD demonstrate increased sympathetic tone and amplified respSNA under baseline conditions and further demonstrate enhanced respSNA and hemodynamic responses to a hypoxic chemoreflex challenge when compared to female Lewis control rats. Moreover, female LPK rats exhibit the same distinctive temporal pattern of respSNA seen in male LPK rats, featuring the peak of respSNA in the PI period and increased inhibition of rSNA during the inspiratory period, with the magnitude of augmentation of respSNA during hypoxia likewise similar (Saha et al., 2019). These results support our hypothesis that CKD-related hypertensive female rats would exhibit heightened sympathetic–respiratory coupling when compared to normotensive controls but do not support our hypothesis that their cardiorespiratory reactivity to chemoreceptor stimulation would be milder when compared to male CKD animals examined under similar experimental conditions.

### Increased SNA

It has been suggested that sympathetic hyperactivity contributes to hypertension in both hypertensive female rat models and women (Hart and Charkoudian, 2014; Maranon et al., 2014). Our demonstration of increased SNA in adult female LPK rats under control conditions corresponds with the marked elevation in blood pressure we observe in these female LPK rats from an early age (Kandukuri et al., 2012; Salman et al., 2015b) and evidence that sympathetic overactivity is a crucial pathological feature in this model, being evident before renal function becomes significantly compromised (Salman et al., 2015b). Current knowledge on the underlying pathogenesis of sympathetic overactivity in female patients with kidney disease, however, is limited.

### Heightened respSNA and Characteristic Temporal Pattern

Our present study demonstrates heightened respSNA in adult female LPK rats in association with hypertension and echoes our data from male juvenile and adult LPK, studied *in situ* and *in vivo*, respectively (Saha et al., 2019). It is also in alignment with studies examining exposure to chronic intermittent hypoxia, where female rats similarly exhibit an augmented respSNA and hypertension (Souza et al., 2016). As demonstrated in male animals (Saha et al., 2019; Toor et al., 2019), in both the female LPK and normotensive control Lewis animals, the peak of respSNA from the onset of the phrenic burst was observed persistently in the PI period under all conditions tested. This is distinct from the findings in male SHR rats, where the peak of respSNA was in the inspiratory phase (Czyzyk-Krzeska and Trzebski, 1990; Simms et al., 2009).

Notably, the temporal pattern of respSNA in female LPK exhibited inspiratory inhibition during control conditions, most apparent in the renal nerve, and this was exaggerated under hypoxic conditions, as we also describe for male animals (Saha et al., 2019). The pattern of respSNA in female LPK rats is therefore not overtly distinguishable to that of male LPK rats, suggesting that sex differences do not exist in the augmented respSNA features of this model of CKD.

### Respiratory Pattern

Exploring the respiratory pattern in the context of augmented respSNA further, we did find in the anesthetized animal that respiratory rate was higher in female LPK rats compared to Lewis controls, as also demonstrated in male LPK rats under control conditions (Saha et al., 2019). Moreover, the duration of PNA was significantly reduced in female LPK rats. This is in contrast to what we saw in male LPK rats, where the duration of the PNA was the same between LPK and Lewis rats. Similarly, in response to chronic intermittent hypoxia, juvenile Wistar female rats exhibited a reduction in inspiratory period compared with control animals (Souza et al., 2016). In contrast, however, juvenile Wistar male rats exposed to chronic intermittent hypoxia showed similar phrenic frequency and duration compared to controls (Zoccal et al., 2008). These findings suggest that sex variations do exist in different phases of the respiratory cycle in disease models, including CKD. Such variation in the respiratory phases between male and female animals is proposed to depend on multiple interrelated inputs at the level of rhythm-generating inspiratory neurons at the brainstem (Garcia et al., 2013, 2016). Therefore, although not examined in this study, it is conceivable that in female LPK rats, the input to respiratory neurons causing variation in the phases of the respiratory cycle may be different from that of male LPK rats.

Intriguingly, our present study also shows that in response to peripheral chemoreceptor stimulation, phrenic frequency was reduced in female LPK rats compared to control rats. This was also reduced in male LPK rats compared to male Lewis rats. These findings are in contrast to the findings of other studies of adult female animals, which demonstrate that respiratory frequency was not changed during acute hypoxia (Garcia et al., 2013) and after chronic intermittent hypoxia (Souza et al., 2015). Given that the study by Garcia et al. (2013) was performed in neonate animals and the work of Souza et al. (2015) in prepubertal animals, the age and stage of development could be one of the reasons for this contrasting result. Different forms of respiratory input to various subclasses of presympathetic motor neurons may therefore trigger this variation in the pattern of respSNA observed during hypoxia in the female LPK rats. Future work is required to explore this possibility.

### Cardiorespiratory Responses to Chemoreceptor Stimulation

In the present study, stimulation of peripheral chemoreceptors using a mild hypoxic stimulus induced an increase in blood pressure and respSNA in both strains, the magnitude of which was significantly greater in LPK rats compared to Lewis. This is consistent with studies that indicate increased sensitivity of peripheral chemoreceptors is a driver of increased sympathetic activity in hypertension and CKD (Hering et al., 2007; Abdala et al., 2012; Paton et al., 2013).

In contrast, hypercapnia produced changes of a similar magnitude between the strains. Thus, in this disease model of CKD, the increase in respiratory sympathetic coupling is not a uniform phenomenon responding to increased chemosensitive drive or increased central respiratory drive (or by corollary, increased breathing effort), but is specific to hypoxic drive and carotid body feedback. Further, although not examined in this study, the augmented response to hypoxia is unlikely to be relayed via central respiratory chemoreceptors such as the retrotrapezoid nucleus neurons, which are an important site of integration between central and peripheral chemoreception (Takakura et al., 2006; Basting et al., 2015; Guyenet et al., 2016). Notably, our findings are comparable to studies in humans that demonstrated specific potentiation of autonomic and ventilatory responses to peripheral chemoreceptor activation in obstructive sleep apnea (OSA) patients using similar isocapnic hypoxia and hyperoxic hypercapnia stimulation paradigms, making the conclusion that tonic chemoreflex activation may contribute to increased sympathetic activity and blood pressure in patients with OSA (Narkiewicz et al., 1998, 1999).

### Changes in Expiratory Phase Activity

There are several classes of presympathetic rostral ventrolateral medulla (RVLM) neurons based on their respiratory modulation, including I activated, PI activated, I inhibited, and non-modulated neurons (Moraes et al., 2013). Interactions between these RVLM presympathetic neurons and respiratory neurons contribute to the phasic respiratory modulation of sympathetic outflow studied in this article (Haselton and Guyenet, 1989; Guyenet, 2006). From studies using male rats, it has been suggested that a hypoxic stimulus with subsequent peripheral chemoreceptor activation drives respiratory neurons and premotor sympathetic neurons to contribute to amplified respSNA during expiration, leading to increased sympathetic drive and hypertension (Zoccal et al., 2008; Zoccal and Machado, 2010; Wong-Riley et al., 2013). In contrast, in studies using female rats, peripheral chemoreceptor stimulation has been shown to drive inspiratory and premotor sympathetic neurons to contribute to amplified respSNA during inspiration, again, however, leading to increased sympathetic drive and hypertension (Souza et al., 2016, 2017). In our present study in female LPK rats, respSNA in the splanchnic nerve under hypoxic conditions showed persistence of excitation in expiration, although renal SNA did not show this expiratory change. This finding contrasts with the finding of male LPK rats, which did not show any excitation of respSNA during expiration.

### Study Limitations

A number of caveats should be considered in the review of the data presented in this article. First, these experiments were performed under urethane anesthetic, which has known impact on central neural control of cardiovascular reflexes (Accorsi-Mendonca et al., 2007), and this is a caveat for all studies performed under these conditions; however, our results are comparable to our studies using the working heart brainstem preparation (Saha et al., 2019), where experiments are performed in the absence of anesthetic, and support translatability of these findings to more physiological conditions. Another consideration is that our sympathetic nerve recording data analysis is reported as absolute value microvolt recordings from multifiber sympathetic nerve preparations. While differences in the contact between the nerve and electrode can result in differences in the microvolt signal amplitude, our work, in this study and previously under both conscious (Salman et al., 2015a) and anesthetized conditions (Yao et al., 2015; Saha et al., 2019), demonstrated SNA at baseline was heightened in the LPK model with a low level of variance within each group of animals. This is consistent with the work of others who similarly report SNA data when comparing baseline activity between different groups of animals (Guild et al., 2010; Stocker and Muntzel, 2013; Burke et al., 2016; Ong et al., 2019) and, importantly, as presented in the seminal study in this field by Simms et al. (2009), who used microvolt data to measure respiratory and sympathetic responses to changes in chemoreceptor stimuli in the SHR rat. Notably, foundational studies that served to establish the high level of SNA in the SHR strain were also analyzed using microvolt data to compare animals across a range of different ages (Judy et al., 1976, 1979). Confidence in our data is further supported by our prior work demonstrating that LPK rats have significantly elevated circulating levels of both norepinephrine and epinephrine (Wyse et al., 2011) and the alignment of our AUC analysis with our μV data in the current study.

## Conclusion

Our present study demonstrates that amplified respSNA is associated with sympathetic overactivity and hypertension in female LPK rats and that peripheral chemoreceptor stimulation provokes a significantly greater increase in respSNA compared to normotensive control animals. Importantly, this fundamental response did not differentiate from our findings in male animals who were studied under similar conditions with a comparable degree of renal dysfunction.

Although hypertension in CKD is a complex and multifactorial disease, in terms of increasing our understanding of the pathophysiology, our results indicate that amplified respSNA is an inherent character of CKD likely driving autonomic dysfunction regardless of sex and highlight that controlling hypertension through reduction of sympathetic activity should be a key focus in the management of this disease. And although we did not delineate the central neural pathways underlying these changes, our findings might be explained in part by tonic activation and increased sensitivity of excitatory chemoreflex afferents. In terms of translational outcomes, this means that future therapeutics directed at reducing SNA, and in turn hypertension, in CKD that target this pathway are applicable to both males and females.

## Data Availability Statement

The original contributions presented in the study are included in the article/Supplementary Material, further inquiries can be directed to the corresponding author/s.

## Ethics Statement

The animal study was reviewed and approved by the Animal Ethics Committee of Macquarie University, NSW Australia.

## Author Contributions

MS was the major contributor to the initial drafting of the manuscript and contributed to experimental design, performed all experiments, analyzed the data, and interpreted results. Q-JS contributed to the experimental work, interpretation of results, and final approval of manuscript. JP and CH contributed to experimental design, analysis, and interpretation of results, drafting of manuscript and final approval. PB contributed to experimental design, interpretation of results, manuscript revision, and final approval. All authors contributed to the article and approved the submitted version.

## Conflict of Interest

The authors declare that the research was conducted in the absence of any commercial or financial relationships that could be construed as a potential conflict of interest.

## Supplementary Material

Data for male animals contained within the Supplementary Material are republished with permission of Elsevier from Saha et al. (2019); permission conveyed through Copyright Clearance Center, Inc.
