# Supplementary material for: Augmented Respiratory–Sympathetic Coupling and Hemodynamic Response to Acute Mild Hypoxia in Female Rodents With Chronic Kidney Disease
Source: Front Physiol. 2021 May 25;12:623599. doi: 10.3389/fphys.2021.623599 (PMC8185289; doi:10.3389/fphys.2021.623599)
Supplement: Supplementary file 1 [file Data_Sheet_1.pdf]

Augmented Respiratory-Sympathetic Coupling and Hemodynamic Response To Acute Mild Hypoxia in Female Rodents With Chronic Kidney Disease

SUPPLEMENTARY MATERIAL

Manash Saha [1,2,3], Qi-Jian Sun [1], Cara M. Hildreth[1], Peter G.R. Burke[1,4] and Jacqueline K Phillips [1*]

[1] Department of Biomedical Sciences, Macquarie University, Sydney NSW, Australia

[2] Department of Nephrology, National Institute of Kidney Disease and Urology, Dhaka, Bangladesh

[3] Graduate School of Medicine, Wollongong University, Wollongong, NSW, Australia

[4] Neuroscience Research Australia, Sydney NSW, Australia

**** Correspondence***

Jacqueline K Phillips

jacqueline.phillips@mq.edu.au

SUPPLEMENTAL TABLE 1 | Renal function parameters in male and female Lewis and Lewis Polycystic Kidney rats.

	Male		Female		P value	
	LPK	Lewis	LPK	Lewis	Sex	Strain
n	8	10	8	9		
Urea (mmol/L)	24.3 ± 2.3*	5.9 ± 0.3	23.7 ± 2.7*	6.7 ± 0.5	0.9259	<0.001
Creatinine (µmol/L)	60.6 ± 11.7*	22.3 ± 4.6	44.7 ± 7.9*	13.1 ± 2.6	0.0869	<0.001
Creatinine Clearance (mL/min)	2.08 ± 0.4*	10.1 ± 2.3	1.5 ± 0.3*	7.4 ± 1.2	0.2782	<0.001

LPK, Lewis Polycystic Kidney; # Significantly different between male vs. female animals within same strain ($P<0.05$), * Significantly different between the strains in same sex ($P<0.05$) as determined using two-way ordinary ANOVA followed by Bonferroni's post-hoc correction. n = number of animals per group. Data for male animals is republished with permission of Elsevier from Saha, M. et al (2019). Respiratory sympathetic modulation is augmented in chronic kidney disease. Respir. Physiol. Neurobiol. 262, 57-66; permission conveyed through Copyright Clearance Center, Inc.

SUPPLEMENTAL TABLE 2 | Effect of peripheral chemoreceptor stimulation on cardiovascular and ventilatory responses in male and female Lewis and Lewis Polycystic Kidney rats

	Male		Female		P value	
	LPK (8)	Lewis (9)	LPK (8)	Lewis (9)	Sex	Strain
Δ MAP (mmHg)	21 ± 5	4 ± 4	30 ± 6	12 ± 6	0.146	0.002
Δ SBP (mmHg)	34 ± 11	5 ± 5	41 ± 8	15 ± 6	0.308	0.001
Δ DBP (mmHg)	16 ± 4	4 ± 4	27 ± 6	9 ± 5	0.135	0.007
Δ PP (mmHg)	18 ± 7	1 ± 2	15 ± 5	5 ± 1	0.9564	0.004
Δ HR (bpm)	11 ± 1	12 ± 2 #	23 ± 3	25 ± 6	0.001	0.832
Δ PNA amplitude (μV)	9.8 ± 3.9	10.9 ± 3.6	11.01 ± 1.9	7.3 ± 1.7	0.682	0.657
Δ PNA duration (sec)	0.07 ± 0.01	0.13 ± 0.02	-0.07 ± 0.03	-0.2 ± 0.03	0.095	0.053
Δ PNA frequency (cycles/min)	-2 ± 2	5 ± 2	-10 ± 3	5 ± 2 *	0.061	<0.001

Delta change in phrenic nerve activity (PNA) and blood pressure (mmHg) under hypoxic or hypercapnic conditions in adult Lewis and Lewis Polycystic Kidney (LPK) rats. MAP: mean arterial pressure, SBP: systolic blood pressure, DBP: diastolic blood pressure, PP: pulse pressure; HR: heart rate. Δ = Delta change in, LPK, Lewis Polycystic Kidney; # Significantly different between male vs. female animals within same strain, * Significantly different between the strains in same sex (*P<0.05*) as determined using two-way ordinary ANOVA followed by Bonferroni's *post-hoc* correction. *n* = number of animals per group. There was significant interaction in only Δ PNA duration (*P<0.05*) between the variables. Data for male animals is republished with permission of Elsevier from Saha, M. et al (2019). Respiratory sympathetic modulation is augmented in chronic kidney disease. Respir. Physiol. Neurobiol. 262, 57-66; permission conveyed through Copyright Clearance Center, Inc.

SUPPLEMENTAL TABLE 3 | Effect of central chemoreceptor stimulation on cardiovascular and ventilatory responses in male and female Lewis and Lewis Polycystic Kidney rats.

	Male		Female		P value	
	LPK (8)	Lewis (9)	LPK (8)	Lewis (9)	Sex	Strain
Δ MAP (mmHg)	16 ± 4	19 ± 3	13 ± 3	9 ± 5	0.123	0.878
Δ SBP (mmHg)	29 ± 8	22 ± 4	20 ± 5	12 ± 6	0.098	0.237
Δ DBP (mmHg)	13 ± 3	18 ± 3	10 ± 2	8 ± 4	0.074	0.643
Δ PP (mmHg)	16 ± 6	4 ± 2	10 ± 3	3 ± 2	0.352	0.026
Δ HR (bpm)	-4 ± 1	-8 ± 1	2 ± 3	-0.3 ± 1	0.002	0.180
Δ PNA amplitude (μV)	9.1 ± 1.6	8.7 ± 2.7	9 ± 2.3	9.4 ± 2.8	0.919	0.992
Δ PNA duration (sec)	0.11 ± 0.02	0.15 ± 0.02#	-0.06 ± 0.02	0.8 ± 0.03*	<0.001	<0.001
Δ PNA frequency (cycles/min)	-4 ± 1	-3 ± 1	-2 ± 1	-0.2 ± 1	0.101	0.365

Delta change in phrenic nerve activity (PNA) and blood pressure (mmHg) under hypoxic or hypercapnic conditions in adult Lewis and Lewis Polycystic Kidney (LPK) rats. MAP: mean arterial pressure, SBP: systolic blood pressure, DBP: diastolic blood pressure, PP: pulse pressure; HR: heart rate. Δ = Delta change in, LPK, Lewis Polycystic Kidney; # Significantly different between male vs. female animals within same strain (*P<0.05*), * Significantly different between the strains in same sex (*P<0.05*) as determined using two-way ordinary ANOVA followed by Bonferroni's *post-hoc* correction. *n* = number of animals per group. There was significant interaction in only Δ PNA duration (*P<0.05*) between the variables. Data for male animals is republished with permission of Elsevier from Saha, M. et al (2019). Respiratory sympathetic modulation is augmented in chronic kidney disease. Respir. Physiol. Neurobiol. 262, 57-66; permission conveyed through Copyright Clearance Center, Inc.

SUPPLEMENTAL TABLE 4 | Effect of peripheral chemoreceptor stimulation on respiratory sympathetic coupling in male and female Lewis and Lewis Polycystic Kidney rats.

	Male		Female		P value	
	LPK	Lewis	LPK	Lewis	Sex	Strain
sSNA (n)	8	9	8	9		
AUC	5.6 ± 2.1	1.8 ± 0.6	8.9 ± 3.4	2.0 ± 0.7	0.257	0.005
PA	3.9 ± 0.9	1.9 ± 0.6	5.5 ± 2.3	1.8 ± 0.6	0.540	0.028
Duration	0.07 ± 0.07	-0.19 ± 0.1	0.21 ± 0.09	0.12 ± 0.07	0.019	0.067
rSNA (n)	5	6	5	6		
AUC	7.5 ± 2.1	3.0 ± 0.8	6.1 ± 1.2	3.1 ± 0.7	0.592	0.006
PA	4.9 ± 1.2	5.2 ± 1.2	4.9 ± 1.2	3.7 ± 0.9	0.562	0.667
Duration	0.13 ± 0.07	-0.09 ± 0.07	0.09 ± 0.1	0.01 ± 0.06	0.689	0.098

sSNA, splanchnic sympathetic nerve activity; rSNA, renal sympathetic nerve activity; AUC, area under curve; PA, peak amplitude; LPK, Lewis Polycystic Kidney; # Significantly different between male vs. female animals within same strain ($P<0.05$), * Significantly different between the strains in same sex ($P<0.05$) as determined using two-way ordinary ANOVA followed by Bonferroni's post-hoc correction. n = number of animals per group. Male animals are the same cohort as reported in Saha, M. et al (2019). Respiratory sympathetic modulation is augmented in chronic kidney disease. Respir. Physiol. Neurobiol. 262, 57-66.

SUPPLEMENTAL TABLE 5 | Effect of central chemoreceptor stimulation on respiratory sympathetic coupling in male and female Lewis and Lewis Polycystic Kidney rats.

	Male		Female		P value	
	LPK	Lewis	LPK	Lewis	Sex	Strain
sSNA (n)	8	9	8	9		
AUC	2.6 ± 0.7	1.0 ± 0.4	2.5 ± 0.9	1.3 ± 0.7	0.921	0.071
PA	2.2 ± 0.5	1.6 ± 0.4	1.7 ± 0.5	2.3 ± 0.7	0.830	0.994
Duration	0.03 ± 0.04	-0.07 ± 0.06	0.1 ± 0.07	-0.09 ± 0.07	0.707	0.024
rSNA (n)	5	6	5	6		
AUC	2.5 ± 1.4	3.2 ± 0.5	4.2 ± 0.9	3.5 ± 1.4	0.360	0.974
PA	1.6 ± 0.7 *	5.7 ± 1.3	1.8 ± 0.5	3.5 ± 0.8	0.308	0.005
Duration	-0.02 ± 0.09	-0.26 ± 0.04	0.2 ± 0.07	0.01 ± 0.09	0.009	0.014

sSNA, splanchnic sympathetic nerve activity; rSNA, renal sympathetic nerve activity; AUC, area under curve; PA, peak amplitude; LPK, Lewis Polycystic Kidney; # Significantly different between male vs. female animals within same strain (*P<0.05*), * Significantly different between the strains in similar sex (*P<0.05*) as determined using two-way ordinary ANOVA followed by Bonferroni's post-hoc correction. n = number of animals per group. Data from male animals is the same cohort as reported in Saha, M. et al (2019). Respiratory sympathetic modulation is augmented in chronic kidney disease. Respir. Physiol. Neurobiol. 262, 57-66.
